# Feasibility of real-time MR thermal dose mapping for predicting radiofrequency ablation outcome in the myocardium in vivo

**DOI:** 10.1186/s12968-017-0323-0

**Published:** 2017-01-25

**Authors:** Solenn Toupin, Pierre Bour, Matthieu Lepetit-Coiffé, Valéry Ozenne, Baudouin Denis de Senneville, Rainer Schneider, Alexis Vaussy, Arnaud Chaumeil, Hubert Cochet, Frédéric Sacher, Pierre Jaïs, Bruno Quesson

**Affiliations:** 1 IHU Liryc, Electrophysiology and Heart Modeling Institute, Foundation Bordeaux University, F-33600 Pessac-Bordeaux, France; 2 Centre de recherche Cardio-Thoracique de Bordeaux, INSERM, U1045, F-33000 Bordeaux, France; 3 Siemens Healthineers France, F-93210 Saint-Denis, France; 4 Image Guided Therapy, F-33600 Pessac, France; 5 Institute of Mathematics of Bordeaux, UMR 5251, F-33400 Talence, France; 6 Siemens Healthineers, Erlangen, Germany; 7 Electrophysiology and Ablation Unit, Bordeaux University Hospital (CHU), F-33600 Pessac, France

**Keywords:** MR thermometry, Radiofrequency ablation, Catheter, Arrhythmia, Cardiac, Electrophysiology

## Abstract

**Background:**

Clinical treatment of cardiac arrhythmia by radiofrequency ablation (RFA) currently lacks quantitative and precise visualization of lesion formation in the myocardium during the procedure. This study aims at evaluating thermal dose (TD) imaging obtained from real-time magnetic resonance (MR) thermometry on the heart as a relevant indicator of the thermal lesion extent.

**Methods:**

MR temperature mapping based on the Proton Resonance Frequency Shift (PRFS) method was performed at 1.5 T on the heart, with 4 to 5 slices acquired per heartbeat. Respiratory motion was compensated using navigator-based slice tracking. Residual in-plane motion and related magnetic susceptibility artifacts were corrected online. The standard deviation of temperature was measured on healthy volunteers (*N* = 5) in both ventricles. On animals, the MR-compatible catheter was positioned and visualized in the left ventricle (LV) using a bSSFP pulse sequence with active catheter tracking. Twelve MR-guided RFA were performed on three sheep in vivo at various locations in left ventricle (LV). The dimensions of the thermal lesions measured on thermal dose images, on 3D T1-weighted (T1-w) images acquired immediately after the ablation and at gross pathology were correlated.

**Results:**

MR thermometry uncertainty was 1.5 °C on average over more than 96% of the pixels covering the left and right ventricles, on each volunteer. On animals, catheter repositioning in the LV with active slice tracking was successfully performed and each ablation could be monitored in real-time by MR thermometry and thermal dosimetry. Thermal lesion dimensions on TD maps were found to be highly correlated with those observed on post-ablation T1-w images (*R* = 0.87) that also correlated (*R* = 0.89) with measurements at gross pathology.

**Conclusions:**

Quantitative TD mapping from real-time rapid CMR thermometry during catheter-based RFA is feasible. It provides a direct assessment of the lesion extent in the myocardium with precision in the range of one millimeter. Real-time MR thermometry and thermal dosimetry may improve safety and efficacy of the RFA procedure by offering a reliable indicator of therapy outcome during the procedure.

**Electronic supplementary material:**

The online version of this article (doi:10.1186/s12968-017-0323-0) contains supplementary material, which is available to authorized users.

## Background

Radiofrequency ablation (RFA) is a well-established procedure to treat cardiac arrhythmias by inducing small thermal lesions in the myocardium. When the area responsible for the arrhythmia is isolated or destructed, durable sinus rhythm is restored and the mechanical function of the heart may even improve in some cases. To prevent recurrence of the arrhythmia, created lesions must be transmural and permanent. Success rate of this procedure is currently limited by a lack of visualization of lesion formation during the procedure [1, 2]. To date, X-Ray fluoroscopy remains the reference imaging modality for navigating catheters in cardiac chambers for electrical mapping and delivery of therapeutic RFA. However, the intrinsic poor soft tissue contrast of this modality does not allow visualization of myocardial injury. Catheter tip temperature rise, changes in tissue electrical impedance and delivered power showed poor correlation with the actual lesion size [3]. On the other hand, an excessive temperature exposure may translate into collateral extra cardiac damages in organs such as lungs, bronchi, phrenic nerves or esophagus [4]. Therefore, there is a need to develop new strategies to offer a more reliable monitoring lesion formation for increased patient safety and improvement of the therapeutic efficiency.

Magnetic Resonance (MR) thermometry using the Proton Resonance Frequency Shift (PRFS) method has been shown to be a relevant technique for the monitoring of the thermal treatment in a number of organs including liver [5, 6], prostate [7], uterus [8] and brain [9, 10]. In addition, it has been demonstrated that MR thermometry-derived cumulative thermal dose (TD) mapping provides a good correspondence between temperature increases for a given time of exposure and the resulting thermal lesion dimensions, using a lethal TD threshold equivalent to a constant heating at 43 °C for 240 min [11].

Recent studies demonstrated the feasibility of cardiovascular MR (CMR) thermometry with electrocardiogram (ECG) and echo-navigator triggering to address challenges inherent to heart contraction and respiratory motion [12–14]. Correction strategies were proposed to compensate for susceptibility artifacts associated with magnetic field changes due to breathing [15, 16]. A spatial resolution of 1.6 × 1.6 × 3 mm^3^ was recently reported with an imaging time of ~100 ms per slice [14]. However, visualization of temperature increase remained limited to a small number of pixels and in vivo data were reported on a limited number of preclinical experiments. To our knowledge, no studies reported correlation between the TD maps derived from MR thermometry and the RFA lesion sizes in the context of the treatment of cardiac arrhythmia.

This study aims at demonstrating the feasibility of simultaneous RF thermal ablation and real-time CMR TD mapping for predicting ablated lesion extent.

## Methods

### Volunteer study

CMR thermometry was evaluated on 5 healthy volunteers under free-breathing conditions. Each volunteer was informed about the protocol and consented to participate to this study.

### Animal study

**Fig. 1:**
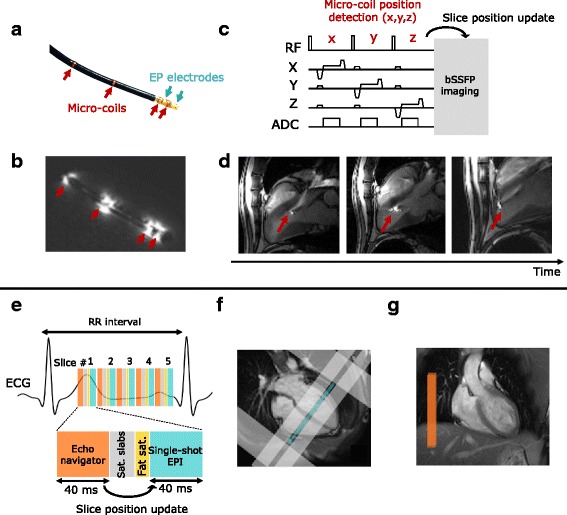
Imaging protocol for catheter navigation (**a**–**d**) and fast CMR thermometry (**e**–**g**) for ablation monitoring. **a** displays photograph of the CMR-compatible catheter equipped with 2 EP electrodes (*blue arrows*) and 4 micro-coils (*red arrows*). EP signals were measured between the two electrodes and the RF current was sent through the tip electrode. The associated magnitude image (**b**) of the catheter in water illustrates the utility of the 4 active markers which appear hyperintense whereas the catheter body stays hypointense. An interactive sequence (**c**) was run during the catheter manipulation. The micro-coil position was detected and used to update the position of the bSSFP imaging slices. Catheter tip (*red arrows*) could be visualized and followed as shown in (**d**). CMR thermometry was performed using a single-shot EPI triggered on ECG (**e**). Up to 5 slices were acquired depending on heart cycle duration. On healthy volunteers, imaging slices were positioned in short axis orientation (4 chambers view **f**, *blue rectangle*) and surrounded by saturation slabs (image **f**, *gray rectangles*): 2 along the FoV in the phase encoding direction to limit aliasing effect and 2 parallels to the imaging slices to reduce the signal of blood. A crossed-pair navigator was positioned on the dome of the liver (image **g**, *orange rectangle* on coronal view) to monitor respiratory motion at the lung/liver interface. The echo-navigator pulse sequence was run before each slice and the detected breathing stage was used to update the position of the following slice

Animal experiments were conducted in vivo on sheep (*N* = 3) weighting 46–54 kg. Anesthesia was induced and maintained with an intravenous injection of ketamine (40 mg/kg/h, IM, Virbac, Carros, France) and 2 mg/kg/h midazolam (Mylan, Canonsburg, PA, U.S.A.). Two MR-compatible catheters were positioned using X-ray angiography system (Toshiba InfiniX, Toshiba Medical, Nasu, Japan). An eight-French MR-compatible catheter (Biosense Webster, Diamond Bar, CA, U.S.A.) was inserted into the right ventricle (RV) for cardiac pacing. A ten-French MR-compatible catheter (MRI Interventions, Irvine, CA, U.S.A.) was positioned into the LV for RFA. This catheter was equipped with 4 MR micro-coils to allow active device tracking under CMR (Fig. 1a red arrows, and b) and with 2 electrophysiological (EP) electrodes (Fig. 1a, blue arrows). The animals were installed in a supine position in the MR scanner and were ventilated using a MR-compatible ventilator (Aestivia, General Electric, Fairfield, CT, U.S.A.) with a respiratory rate of 15 breaths per minute (bpm). Vital signs including cardiac rhythm, intra-arterial pressure, capnometry and rectal temperature, were monitored during all the experiments (Carescape, General Electric, Fairfield, CT, U.S.A.).

### Radiofrequency ablation device

The LV catheter was connected to a clinical RF generator (Stockert Medical Solution, Freiburg, Germany) located outside the Faraday cage, using the ClearTrace system [17] (MRI Interventions, Irvine, CA, U.S.A.). A RF current at 500 kHz was sent between the catheter tip electrode and an adhesive return electrode (MONOPlate, ERBE, Tübingen, Germany) positioned on animal skin. Electrical isolation between RF generator and MR scanner was achieved by low pass filters introduced in the transmission line [13] to prevent RF artifacts on MR acquisitions due to possible interferences between RFA device and MR receivers. The catheter was irrigated with a saline solution (0.9% concentration) with a flow rate of 3 mL/min during all the procedure and 10–12 mL/min during RFA. The heart was paced with the catheter located into the RV to reduce the risk of inducing ventricular arrhythmia commonly observed in large animal models during RFA. EP signals were measured between the tip electrode and the 2nd electrode of catheters in both ventricles and displayed on a clinical EP acquisition system (Bard, LabSystem PRO, Boston Scientific, Marlborough, MA, U.S.A.). A numerical low-pass Butterworth filter (cutoff frequency = 30 Hz, 8th order) was applied to LV signal for denoising.

### Imaging protocol

CMR was performed on a 1.5 T MR system (Avanto, Siemens Healthcare, Erlangen, Germany) using two clinically used 16-channel cardiac coils (Invivo Corporation, Gainesville, FL, U.S.A.). After scout views were acquired, the imaging protocol was set as follow:

In volunteers, the receiver coils were positioned anterior and posterior around the chest. CMR temperature images were acquired in short axis orientation under free breathing.

In animals, the receiver coils were positioned left and right to the chest. Catheter navigation was performed under CMR guidance to position the catheter at different locations within the LV. Then, CMR temperature images were acquired under assisted ventilation during RF ablation. 3D T1-weighted images were acquired after completion of the ablations to assess lesion sizes. Details on each acquisition sequence are provided below.

#### Catheter navigation

Real-time guidance of the catheter was performed using an interactive sequence made of a balanced steady state free precession (bSSFP) pulse sequence (TE/TR = 1.93/183 ms, resolution = 1.8 × 1.8 × 4 mm^3^, Field of view (FoV) = 225×225 mm^2^, Flip angle (FA) = 45°) interleaved with a catheter tracking module (BEAT IRTTT: Interactive Real Time Tip Tracking, Siemens Healthcare, Erlangen, Germany). This module consisted in a 3D encoding of the micro-coils position (Fig. 1c) along three successive gradient orientations with a total duration of 20 ms. The 3D position of the catheter coils was computed online and three slices were automatically repositioned accordingly to align the center of the slice on the catheter tip (Fig. 1d). Images were displayed on a remote monitor inside the CMR room to provide visual feedback to the operator.

#### Dynamic temperature mapping

A fat-saturated, single-shot Echo Planar Imaging (EPI) sequence was combined with GRAPPA parallel acquisition technique (acceleration factor of 2) to achieve a 1.6×1.6×3 mm^3^ spatial resolution, TE/TR/FA = 18–20 ms/110 ms/60°, bandwidth = 1576 Hz/px, FoV = 180×180 mm^2^, 6/8 partial Fourier. Up to five slices (spacing between slices = 0.3 mm) were acquired at each heartbeat during 250–300 consecutive cardiac cycles with ECG triggering (Fig. 1e). Saturation slabs were positioned along the FoV in the phase encoding direction to avoid aliasing and two additional saturation bands were set parallel to the imaging slices to reduce the signal of blood inside the cavities (Fig. 1f), as proposed in [18]. A crossed-pair navigator was positioned on the dome of the liver to adjust the slice position (with 0.6 tracking factor) in real-time for compensating through-plane respiratory motion (Fig. 1g) [19]. On volunteers, the group of slices was positioned in short axis orientation at the medium part of the heart to evaluate the precision of the method on both ventricles. On sheep, the group of slices was positioned accordingly to intersect the catheter tip, previously visualized during the MR-guided navigation. Before the RFA, a series of 70 repetitions was first acquired to assess the CMR thermometry stability in the myocardium surrounding the catheter tip. Then an identical EPI acquisition was run for 250–300 repetitions and RF energy was started at the 50th image.

### MR thermometry pipeline

Acquired EPI raw data were streamed online via TCP/IP to a remote computer for image reconstruction using the Gadgetron framework [20]. Reconstruction pipeline included EPI ghost-correction using three central line of k-space, followed by GRAPPA parallel reconstruction [14]. Zero filling was applied prior to Fourier transform of data, resulting in a matrix size of 224×224 px and a reconstructed pixel size of 0.8×0.8 mm^2^ in plane.

The PRFS thermometry method computes temperature change ΔT from difference between phase image *φ*_*t*_ and a reference phase image *φ*_*ref*_ acquired prior to heating:

$$ \Delta \mathrm{T}=\left({\upvarphi}_{\mathrm{t}}-{\upvarphi}_{\mathrm{ref}}\right)\cdot \mathrm{k}\kern3.25em \mathrm{with}\kern0.5em \mathrm{k}={\left(\frac{\upgamma}{2\uppi}\cdot \upalpha \cdot {\mathrm{B}}_0\cdot \mathrm{T}\mathrm{E}\right)}^{-1} $$

Where *γ* is the gyromagnetic ratio (=42.58 MHz/T), *α* is the PRF temperature coefficient (= − 0.0094 ppm/°C), *B*_0_ is the magnetic field strength (=1.5 T) and TE is the echo time. The use of the PRFS technique on moving organs such as the heart requires motion and associated susceptibility artifacts correction.

#### Motion correction

Through-plane respiratory motion was compensated by a real-time update of slice position from the echo-navigator readings measured at the liver-lung interface. A Principal Component Analysis (PCA)-based optical-flow algorithm was used to estimate residual in-plane motion on magnitude images [21]. Phase images were registered at a fixed position using the calculated motion fields.

#### Correction of respiratory-induced phase variations

Due to magnetic field variations associated with respiration, susceptibility artifacts induce variations on phase images. Thus direct computation of temperature maps from registered phase images may lead to significant errors. A PCA-based method was performed to build a parameterized flow model that establishes a relationship between motions fields and phase variations over the 30 first repetitions of the time series (learning step). This model was then used to correct artifacts of the following repetitions during the interventional procedure. This correction method was introduced in an earlier study [22].

Phase drift compensation was also performed to minimize the effect of fluctuations of magnetic field induced by the heating of magnet components during EPI scanning [14, 23]. Finally, temperature maps were temporally filtered on a pixel-by-pixel basis with a low-pass Butterworth filter (cutoff frequency of 0.14 Hz), taking advantage of the high temporal resolution (<1 s) compared to the slow evolution of the temperature in tissue.

To assess the thermometry precision, the temporal average μ_T_ and the temporal standard deviation σ_T_ were computed on each pixel over ~2 min 30 s of acquisition without heating on healthy volunteers. Regions of interest (ROIs) were hand-drawn around the LV and the RV to perform statistical analysis.

#### Thermal dose mapping

First introduced by Sapareto et al. [11], cumulative TD was formulated in each pixel as follows:

$$ T D=\left\{\begin{array}{c}\hfill {\displaystyle {\int}_0^t2\left(\mathrm{T}\left(\mathrm{t}\right)-43\right)\mathrm{dt}\kern1.5em \mathrm{if}\kern0.5em \mathrm{T}\left(\mathrm{t}\right)>43{}^{\circ} C}\hfill \\ {}\hfill {\displaystyle {\int}_0^t4\left(\mathrm{T}\left(\mathrm{t}\right)-43\right)\mathrm{dt}\kern1.5em \mathrm{if}\kern0.5em \mathrm{T}\left(\mathrm{t}\right)<43{}^{\circ} C}\hfill \end{array}\right. $$

Where T(t) = T_ref_ + ΔT is the absolute temperature at the time t. Rectal temperature served as a reference temperature value T_ref_. The dose corresponding to a constant heating at 43 °C for 240 min was considered as the lethal threshold. Computed TD maps were corrected to take in account the uncertainty of the temperature measurement σ_T_, using the following formula [24]:

$$ {\mathrm{TD}}_{corrected}\left(\mathrm{x},\mathrm{y}\right)=\mathrm{T}\mathrm{D}\left(\mathrm{x},\mathrm{y}\right)\cdot {e}^{-0,5\left( \ln (2)\cdot {\upsigma}_T\left( x, y\right)\right){}^2} $$

Where σ_T_ is the temperature standard deviation map, calculated on each pixel before RFA over 20 repetitions (~20 s). Computed temperature and TD maps were sent in real-time to a dedicated thermometry software (Thermoguide, Image Guided Therapy, Pessac, France) for display and online monitoring of the ablation.

### Post-ablation lesion visualization

After each RFA, a 3D navigator-gated Turbo Flash IR pulse sequence was performed with long inversion time (TI) for blood suppression in order to visualize the lesion without the use of contrast agents, as proposed by M. Guttman et al. [25]. The parameters of the sequence were: TI = 700 ms, resolution = 1.3×1.3×2.5 mm^3^, FoV = 360×360 mm^2^, matrix = 288×288 px, ECG-triggered every two heartbeats, FA = 11°, 31 segments, 60 slices. The contrast-to-noise ratio (CNR) was measured as the inner lesion signal intensity minus surrounding tissue signal intensity divided by the standard deviation of the noise measured in a ROI located outside the body (Syngo.via software package, Siemens Healthcare, Erlangen, Germany). Apparent lesion sizes measured on the resulting images will be referred as 3D T1-w dimensions.

### Macroscopic examinations

After completion of the MR-guided RFA and 3D imaging, the animals were euthanized with a lethal intravenous dose of Dolethal (Vetoquinol, Lure, France) until complete cardiac arrest was attested. The heart was rapidly excised and dissected to expose each ablation lesion. Each lesion sample was stained at 37 °C with tetrazolium chloride (TTC) solution to assess the range of ablation area. Lesions were measured with a ruler and dimensions were correlated to dimensions observed on 2-dimensional (2D) TD maps and on 3D T1-w images.

### Lesion size measurements

To compare 2D TD map and 3D T1-w lesion sizes, the equivalent 2D slice of the CMR thermometry sequence displaying the largest TD dimensions was reconstructed from the 3D T1-w volume. Largest and smallest dimensions of the apparent lesion were measured on both images. To compare 3D T1-w and macroscopic lesion sizes, 2 slices were reconstructed from the 3D volume to approach the orientation of the heart sample dissection. Three measurements were performed: largest and smallest dimensions from endocardium side and depth into the myocardium.

## Results

### Volunteer study

**Fig. 2:**
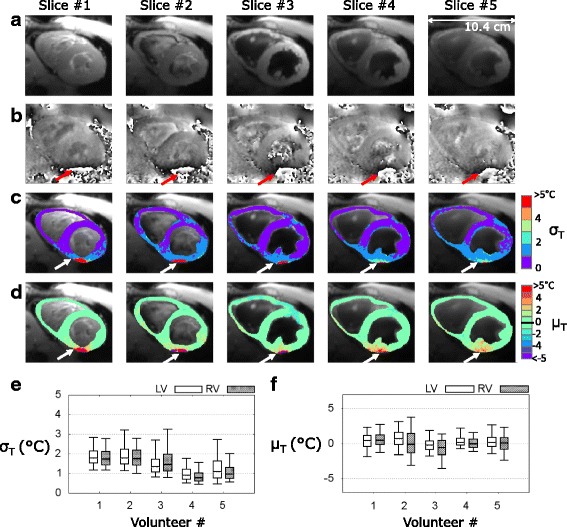
CMR thermometry evaluation on 5 healthy volunteers in short axis view. Typical results obtained on a volunteer (#5) are displayed: **a** averaged registered magnitude over 2 min 30 s of acquisition and (**b**) phase image presenting aliasing at the heart-liver-lung interface (*red arrows*). Temporal standard deviation σ_T_ (**c**) and mean μ_T_ (**d**) temperature maps are overlaid on averaged magnitude in a handmade ROI surrounding the all myocardium. *White arrows* indicate degraded CMR thermometry performance induced by remaining phase wraps. Pixels of this area with aberrant results (σ_T_ > 7 °C) were excluded from the statistical analysis presented in (**e**-**f**). Box-and-whisker plots of standard deviation σ_T_ (**e**) and mean μ_T_ (**f**) of the distribution of temperature in handmade ROIs surrounding the LV and the RV. For each graph, the levels of temperature correspond to 10% (*lower horizontal bar*), 25% (*lower limit of the box*), median value (*horizontal line inside the box*), 75% (*upper limit of the box*) and 90% (*upper horizontal bar*) of the distribution

The rate and maximal amplitude of breathing were 24 ± 2 bpm and 9.2 ± 4.2 mm, respectively. The use of saturation slabs apart the imaging slice group resulted in effective blood suppression in three out of five slices, as shown in Fig. 2a on averaged magnitude images. Phase images showed significant phase wraps in all volunteers at the heart-liver-lung interface, depicted by the red arrows in Fig. 2b. The results of the evaluation of the accuracy σ_T_ and average of temperature μ_T_ in the myocardium of one typical volunteer are shown as an overlay on the averaged registered magnitude images in Fig. 2c and d, respectively. A reduced temperature precision was observed in the area with remaining phase wraps. The statistical analysis of temperature distribution over all volunteers is presented in Fig. 2 (e-f) using box and whisker plots for LV and RV ROIs. Over all the volunteers, the standard deviation of temperature σ_T_ for 2 min 30 s was 1.5 ± 0.4 °C in LV and 1.5 ± 0.5 °C in RV, excluding pixels where the thermometry performance was too much degraded (σ_T_ >7 °C), representing 3.7 ± 0.9% of the pixels of the myocardium (627 ± 240 pixels were excluded over a total of 16×10^4^ ± 3.2×10^3^ pixels). The temporal average was 0.3 ± 0.3 °C in the LV and 0.7 ± 0.5 °C in the RV, illustrating the efficiency of phase drift correction (up to 5 °C over 2–3 min of dynamic acquisition) of the MR thermometry pipeline. Thickness of heart walls measured on conventional cine images (resolution 1.5×1.5×7 mm^3^, 25 phases/cardiac cycle with retrospective reconstruction) at the medium part of the heart were 4.3 ± 0.7/2.7 ± 1.3 mm (mean ± sd in Systolic/Diastolic phases) in RV and 11.6 ± 3.4/7.4 ± 2.1 mm in LV.

### In vivo radiofrequency ablations monitoring

For each animal, the MR-compatible catheter could be successfully navigated and visualized at a frame rate of 3 Hz, with automatic realignment of the imaging stack on the catheter tip. The catheter could be repositioned to a new location in the LV in less than 5 min. 12 RF procedures were performed at different locations in the endocardium of the 3 sheep. The standard deviation of the temperature σ_T_ before RFA was 1 °C or below on the LV. Catheter appeared as a hypointense area with a larger diameter (6 mm) than the actual catheter dimension (3 mm). However, thermometry remained precise enough to monitor the temperature evolution in surrounding myocardial tissue.

**Fig. 3:**
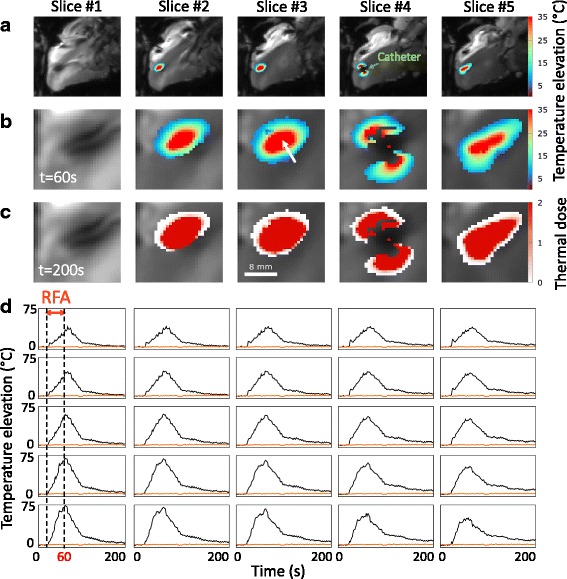
Real-time CMR thermometry during RFA in vivo on a sheep LV. Dynamic CMR thermometry was performed in real-time during a RFA (70 W for 40 s, RFA #4 on sheep #3). **a** Temperature maps at *t* = 60 s, corresponding to 40 s of RF delivery, are overlaid on averaged registered magnitude images within a hand-drawn ROI surrounding the heating zone. Heating zone and associated TD (*t* = 200 s) are zoomed on (**b**) and (**c**) in a 30x30 pixels ROI. The value 1 refers to one time the lethal TD threshold equivalent to 43 °C for 240 min. **d** Temperature evolution in time in 5 × 5 pixels of slice #3 centered on the *white arrow*. *Orange line* depicts baseline temperature in a single pixel outside heating zone. (Additional file 1: Figure 3 shows CMR thermometry evolution every 4 dynamic repetitions)

Figure 3 presents typical CMR thermometry results of a radiofrequency application at 70 W for 40 s. The local temperature evolution around the catheter tip showed a progressive rise during energy delivery with a maximal relative temperature increase of 75 °C on the central slice, followed by tissue cooling after completion of RF delivery (Fig. 3a, zoom on heating zone in Fig. 3b). TD maps were computed online with the lethal dose colored in red (Fig. 3c, the lethal threshold corresponds to 1). The temperature increase in 5×5 pixels centered on heating spot is displayed in Fig. 3d.

**Fig. 4:**
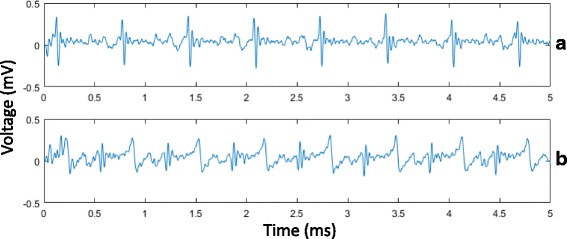
Endocardial bipolar signal recorded at the tip of the MR-compatible ablation catheter in the LV of a sheep: **a** before the ablation and **b** after the ablation, showing voltage abatement

ECG recording from the ablation catheter could be performed during the procedure. As displayed on Fig. 4, changes in ECG signals acquired post-ablation (Fig. 4b) differed from the signals observed before the procedure (Fig. 4a). During the ablation, the simultaneous CMR thermometry acquisition caused significant artifacts and ECG signals were barely readable during the RFA.

Table 1 reports CMR thermometry results of 12 RF procedures performed on 3 sheep. The 12 RF procedures resulted in induction of a thermal lesion and were successfully monitored by CMR thermometry and dosimetry. Remaining spatial phase wraps at the apex prevented efficient CMR thermometry for one ablation that was not reported on Table 1. An average of 59.2 ± 7.9 W of RF power was delivered for 72.5 ± 18 s, leading to a total RF energy delivery ranging from 2.8 to 7.0 kJ (mean ± sd = 4.3 ± 1.3 kJ).

**Table 1:** In vivo CMR thermometry results on 3 sheep. Parameters of 12 RFA are reported in this Table: the power, the duration and the energy of the RF delivery, the maximal temperature reached in the LV and the 2D dimensions of each lesion created

Lesion sizes in 2D							
Sheep #	RFA #	Power (W)	Dur. (s)	Energy (J)	Temp max (°C)	TD map (mm^2^)	T1-w (mm^2^)
1	1	60	60	3600	30 °C	9×4	9×5
	2	60	90	5400	55 °C	6.5×4	7×4
	3	70	100	7000	30 °C	8×3	9×3
	4	70	90	6300	40 °C	7.5×5	6.5×4
2	1	60	60	3600	40 °C	11×4	11×5
	2	60	60	3600	45 °C	12.5×6	11×5.5
	3	50	90	4500	35 °C	5×8	6×6
	4	60	60	3600	45 °C	10×6	9×5
3	1	50	90	4500	55 °C	12×10	10×7
	2	50	60	3000	30 °C	8×8	8×6
	3	50	70	3500	55 °C	10.5×7	10×7
	4	70	40	2800	75 °C	10×7	10×7

Lesion extent maximal dimensions were measured in 2D on the cumulative TD maps and on the equivalent slice of the 3D T1-w volume

### Post-ablation lesion size measurements

**Fig. 5:**
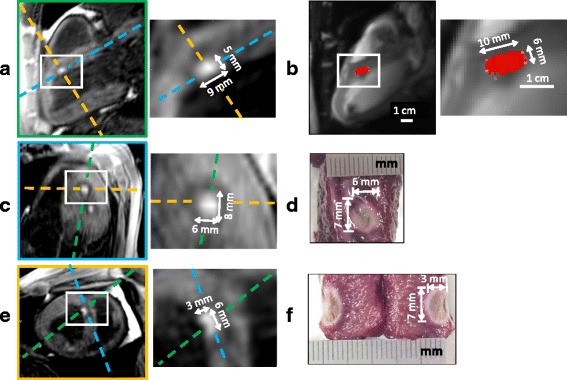
Comparison of lesion dimensions of one representative RFA between post-ablation T1-w 3D images (**a**, **c** and **e**), real-time TD map (**b**) and macroscopic observations with TTC staining (**d** and **f**). Cumulative TD was computed in real-time simultaneously to a RFA of 60 s at 60 W (RFA #4 on sheep #2), given the TD map (**b**) at the end of the procedure. After the ablation, a 3D T1-w IR volume was acquired and the created lesion could be visualized in hyper signal compared to the surrounding tissue attributed to edema. To compare 2D TD map and 3D-IR lesion sizes, the equivalent 2D slice was reconstructed from the 3D T1-w volume, resulting in the image (**a**, *green dashed line* perpendicular to other views **c** and **e**) with the same position, orientation and zoom than (**b**). After the intervention, the animal was euthanized and the heart was dissected to expose the lesion. The macroscopic view from the endocardium side is shown in (**d**) and the equivalent slice (**c**) was reconstructed from the 3D T1-w volume (*blue dashed line* perpendicular to other views **a** and **e**). The lesion sample was finally cut to expose the extent in depth in the myocardium (**f**) and the equivalent slice (**e**) was reconstructed from the 3D T1-w volume (*yellow dashed line* perpendicular to other views **a** and **c**)

**Fig. 6:**
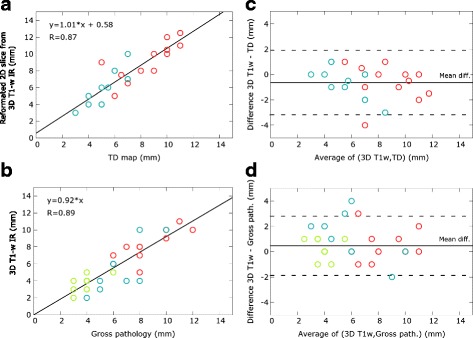
RF lesion dimensions (*N* = 12 lesions) correlation between measurements on real-time thermal dose maps, post-ablation T1-w images and gross pathology. Pearson’s linear correlation on (**a**) reformatted 2D slices from T1-w IR images and TD maps (2 dimensions per lesion, *N* = 24), and (**b**) T1-w IR images and macroscopic measurements (3 dimensions per lesion, *N* = 36). Dimensions are colour-coded: *red* for largest dimensions, *blue* for smallest dimensions and *green* for depth dimensions (**b** and **d** graphs only). It must be noted that several points correspond to more than one measure, since some lesions had identical dimensions. Pearson’s correlation coefficient R and coefficients of the linear regression are indicated on the graphs. Bland and Altman representation of the agreement between the lesion dimensions of the T1-w IR images and TD maps (**c**) and between the T1-w IR images and gross pathology measurements (**d**). *Dashed lines* indicate 95% confidence limits

Figure 5a displays typical lesion visualization on 2D slice reconstructed from the 3D T1-w volume after a RFA, together with the cumulative TD map (Fig. 5b) obtained in real-time during the ablation. The 3D T1-w images obtained with an IR pulse sequence with a TI of 700 ms allowed visualization of lesion core in hyper signal with a CNR of 88.8 ± 38.3 with the surrounding tissue. The volume was acquired after each new ablation with an acquisition time in the range of 15 min to 25 min, depending on the acceptance rate of the echo-navigator and the heart cycle duration. The dimensions of this lesion performed at 60 W for 60 s were 9x5 mm^2^ on T1-w image and 10x6 mm^2^ on TD map. Correlation between ablation size on T1-w images and TD maps is shown in Fig. 6a with a Pearson’s coefficient *R* = 0.87 and a linear regression slope of 1.01 and an off-set of 0.58 mm. In Fig. 5 are presented T1-w volume (Fig. 5c) and macroscopic views (Fig. 5d) of the same representative lesion for purpose of comparison. The three measured dimensions were 8×6×3 mm^3^ and 7×6×3 mm^3^, respectively. Correlation study between T1-w volume and macroscopic measurements is presented Fig. 6 b with a Pearson’s coefficient R = 0.89 and a linear regression slope of 0.92 and an off-set of 0.00 mm. The Bland-Altman analysis displayed in Figs. 6c and d showed no bias depending on the ablation size. Only five measurements were found outside the 95% confidence interval. 2D lesion size estimation from TD maps was found larger than T1-w images measurements: 0.63 ± 1.3 mm (Fig. 6c) and 3D lesion size measurements on T1-w volume was found larger than macroscopic measurement: 0.46 ± 1.2 mm (Fig. 6 d). No correlation analysis could be performed between 2D TD maps and macroscopic measurements since no reliable 3D correspondence could be performed between orientation/position of 2D CMR slice and gross pathology dissection.

## Discussion

One major challenge of RFA for the treatment of cardiac arrhythmia is the ability to visualize the lesion formation in the myocardium in real-time. Objectives of this mini-invasive therapy are to achieve a transmural ablation of the arrhythmogenic substrate while preserving the surrounding healthy tissues and to create a continuous electrical isolation through multiple adjacent RFA performed sequentially. This study demonstrates that i) CMR thermometry using our pipeline allows real-time tissue temperature monitoring, including during RF delivery, ii) thermal dosimetry predicts myocardial lesion size. To achieve this goal, we developed a rapid (~110 ms/image), multi-slice echo-navigated CMR thermometry method with a spatial resolution in the range of one millimeter, including real-time motion correction and compensation of associated susceptibility artifacts on phase images.

Compared to the recent paper from Ozenne et al. [14], an echo-navigated slice following technique was implemented to compensate for respiratory motion in free-breathing conditions, providing full flexibility in the slice orientation. This improvement allowed for the evaluation of CMR thermometry in five volunteers in short axis orientation and resulted in an uncertainty of approximately 1.5 °C in the myocardium over 2.5 min of acquisition. Although the wall thickness is thinner in the RV than in the LV, similar temperature precision could be achieved in both ventricles. To our knowledge, this is the first study to report temperature measurements in the RV. The echo-navigator based slice following approach was first reported by Denis de Senneville et al. [13] for CMR thermometry. However, the proposed acquisition protocol suffered from an insufficient spatial resolution (voxel size of 2.5x2.5x6 mm^3^) to precisely monitor RFA and temperature measurements in the RV. Moreover, only two RFA were performed in vivo in animals.

In this work, lesion sizes measured on TD, 3D T1-w imaging and gross pathology from 12 RFA performed in 3 sheep were compared. One procedure did not produce a lesion that was attributed to a poor electrode-tissue contact. The results showed an excellent correlation (slopes close to unity and off-set lower than 1 mm) between apparent lesion sizes measured in 2D on TD maps vs T1-w images and in 3D on T1-w images vs macroscopic measurements. Differences between lesion sizes remained in the range of image spatial resolution (0.8 mm). Thus, TD mapping can be used as a reliable surrogate for assessing lesion size in real-time during RFA. However, direct comparison between TD maps and gross pathology was not performed since the slice thickness of thermometry sequence (3 mm) was considered too large to assess the TD deposition in 3D with a sufficient precision, regarding the lesion size dimensions reported in Table 1.

Interestingly enough, this study shows that identical RFA parameters (Table 1, duration and power of RF delivery) can lead to different thermal lesion sizes. This may be explained by a number of factors such as the quality of the contact between the catheter tip and the tissue, potential local differences in tissue electrical and thermal conductivities, influence of blood flow, and efficiency of catheter cooling. This has been suspected clinically but the current method now provides an accurate and reliable way to directly measure the actual temperature in the myocardium and accumulated thermal dose, and thereby guide RF delivery in a much more effective way to achieve the desired endpoint [26]. This strategy should be compared to indirect means such as lesion index algorithm using contact force information.

Kolandaivelu et al. [12] proposed to use an isotherm lethal threshold of 50 °C assuming no time-temperature relation. They studied the correlation of the transmurality of the lesions (depth in short axis orientation) between MR thermometry, LGE MRI and gross pathology over 6 RF ablations in dogs. They reported that lesion transmurality by thermography was within 20% of that measured by pathology and LGE MRI. However, the insufficient temporal resolution of 10 to 20 s per image associated with breath-holding prevented an accurate and continuous monitoring of the temperature evolution. Moreover, such an approach remains hardly applicable to patients. Visualization of lesion using LGE CMR was found to overestimate the pathological lesion size [12, 27]. In the present study, we performed 3D IR T1-w imaging to visualize the lesions after the procedure without injection of contrast agent. The lesions appeared as an area of hyperintensity, well demarcated (CNR = 88.8 ± 38.3) from the surrounding edema appearing as hypointense [28] and healthy tissue. Image contrast did not change significantly (data not shown) over the time course of the experiment (>3 h). These findings were consistent with results obtained by other groups with the same CMR acquisition pulse sequence [25, 29]. This method offers the advantage of providing a high CNR without injection of a contrast agent, allowing to visualize previously created lesions at once. This is of particular interest to detect potential gaps between successive RFA that can result in arrhythmia recurrence. Redo procedure to create a continuous lesion can therefore be facilitated without requiring to wait for contrast agent elimination [27]. However, the sequence remained relatively time consuming (~15-25 min depending on the heart rate and the breathing cycle duration) and should not be used too often to avoid increasing total procedure time. Recent improvement in acceleration techniques, such as iterative reconstruction [30, 31], could help in reducing the acquisition time for potential clinical practice.

### Limitations

This study aimed to investigate if the thermal dose can predict the actual lesion size. However, it was not designed to demonstrate that CMR thermometry can guide RF delivery in order to achieve transmural and contiguous lesions that are mandatory for efficient curative treatment of arrhythmia. As a consequence and considering this study as a first step, we chose to perform several ablations at distinct locations within the LV, to allow to characterize each RF delivery without bias from overlapping lesions.

Although reliable thermometry stability was achieved in 96% of the voxels located on the myocardium, the region near the liver-heart-lung interface remained prone to stronger spatial and temporal susceptibility variations that lead to higher temperature standard deviation σ_T_ (>7 °C)_._ Such an observation was already reported in previous studies [13–15]. Further improvement in image processing might compensate for this artifact to retrieve accurate temperature measurements in this area.

The proposed CMR thermometry method needs to be evaluated on patients suffering from cardiac arrhythmia. Under these conditions, synchronization between the image acquisition and the ECG may be prone to errors and increased temperature uncertainty. Unobserved cardiac contraction pattern during the learning step may be difficult to compensate and justify further investigations and optimization of correction algorithms.

Echo-navigator based slice tracking was assumed to fully correct for through-plane respiratory motion. However, free-breathing patients could present more erratic respiratory motion than healthy volunteers and mechanically ventilated animals. The performance of the slice following should be evaluated under such conditions. The implementation of simultaneous active catheter tracking [32, 33] and RFA could address this limitation with a slice always positioned on the catheter tip during CMR thermometry.

The first results of thermometry stability on the RV of healthy volunteers are promising for future monitoring of RFA in this ventricle. However, the spatial resolution remains insufficient for atrial RFA considering the thickness of the atrial wall (~2-3 mm). Recently, the use of endovascular coils embedded in the catheter was proposed and may be explored to achieve high spatial resolution [34, 35] for monitoring temperature change in the atria.

The current MR-compatible catheters did not allow precise EP recording during RFA during CMR, due to perturbation induced by CMR gradient switching. Only two contact EP electrodes were available close to the catheter tip due to the presence of MR receiver coils, providing limited contact EP measurements. Thus, improvement of catheter design and associated instrumentation is also mandatory before clinical evaluation.

## Conclusion

This study demonstrates that reliable thermometry can be achieved online on both ventricles using a multi-slice fast imaging sequence combined with slice tracking for respiratory motion compensation. In addition, the thermal dose mapping is highly correlated with post-ablation 3D T1-w images that were also correlated with actual lesion sizes measured at gross pathology. This real-time thermal dosimetry may provide guidance during RF delivery and therefore improve safety and efficacy of catheter ablation.

## Additional file

Additional file 1: Figure 3. Animation of real-time CMR thermometry during RFA in vivo on a sheep LV (update every 4 dynamic repetitions). Dynamic CMR thermometry was performed in real-time during a RFA (70 W for 40 s, RFA #4 on sheep #3). A) Temperature maps at t = 60 s, corresponding to 40 s of RF delivery, are overlaid on averaged registered magnitude images within a hand-drawn ROI surrounding the heating zone. Heating zone and associated TD (t = 200 s) are zoomed on (B) and (C) in a 30x30 pixels ROI. The value 1 refers to one time the lethal TD threshold equivalent to 43 °C for 240 min. D) Temperature evolution in time in 5x5 pixels of slice #3 centered on the white arrow. Orange line depicts baseline temperature in a single pixel outside heating zone. (GIF 10160 kb)

## Abbreviations

2D: 2-dimensional
3D: 3-dimensional
bSSFP: Balanced steady state free precession
CMR: Cardiovascular magnetic resonance
CNR: Contrast to noise ratio
ECG: Electrocardiogram
EP: Electrophysiology
EPI: Echo Planar Imaging
FA: Flip angle
FoV: Field of view
IR: Inversion recovery
IRTTT: Interactive real time tip tracking
LGE: Late Gadolinium Enhancement
LV: Left Ventricle
MR: Magnetic resonance
MRI: Magnetic resonance imaging
PCA: Principal component analysis
PRFS: Proton resonance frequency shift
RFA: Radiofrequency ablation
ROI: Region of interest
RV: Right ventricle
T1-w: T1 weighted
TCP/IP: Transmission Control Protocol/Internet Protocol
TD: Thermal dose
TE: Echo time
TI: Inversion time
TR: Repetition time
TTC: Tetrazolium chloride
