# Supplementary figures and images for: Feasibility of real-time MR thermal dose mapping for predicting radiofrequency ablation outcome in the myocardium in vivo
Source: J Cardiovasc Magn Reson. 2017 Jan 25;19:14. doi: 10.1186/s12968-017-0323-0 (PMC5286737; doi:10.1186/s12968-017-0323-0)

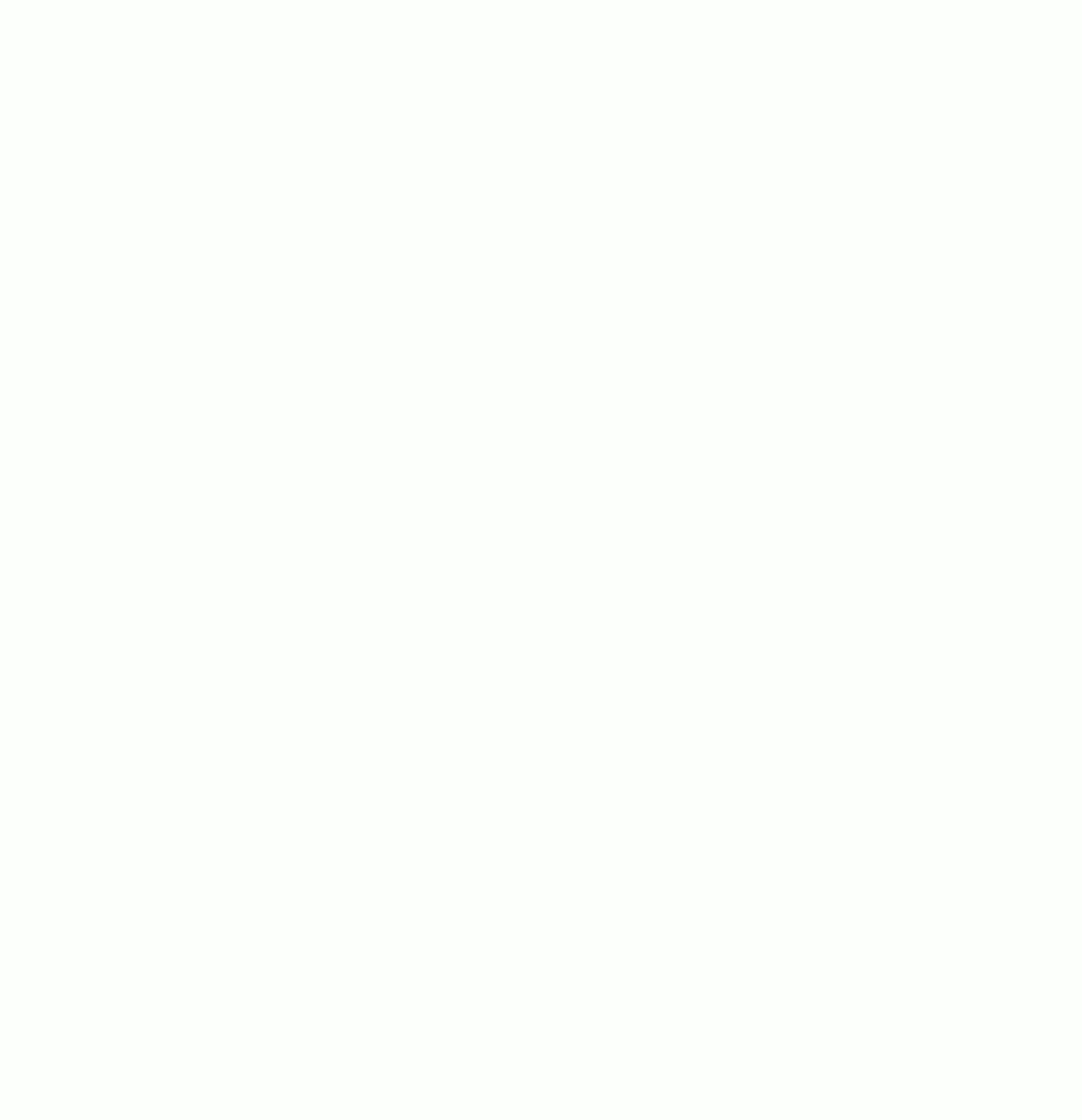

Supplement: Additional file 1: Figure 3. — Animation of real-time CMR thermometry during RFA in vivo on a sheep LV (update every 4 dynamic repetitions). Dynamic CMR thermometry was performed in real-time during a RFA (70 W for 40 s, RFA #4 on sheep #3). A) Temperature maps at t = 60 s, corresponding to 40 s of RF delivery, are overlaid on averaged registered magnitude images within a hand-drawn ROI surrounding the heating zone. Heating zone and associated TD (t = 200 s) are zoomed on (B) and (C) in a 30x30 pixels ROI. The value 1 refers to one time the lethal TD threshold equivalent to 43 °C for 240 min. D) Temperature evolution in time in 5x5 pixels of slice #3 centered on the white arrow. Orange line depicts baseline temperature in a single pixel outside heating zone. (GIF 10160 kb) [file 12968_2017_323_MOESM1_ESM.gif]
